# Research Progress, Challenges, and Breakthroughs of Organoids as Disease Models

**DOI:** 10.3389/fcell.2021.740574

**Published:** 2021-11-16

**Authors:** Yisheng Huang, Zhijie Huang, Zhengming Tang, Yuanxin Chen, Mingshu Huang, Hongyu Liu, Weibo Huang, Qingsong Ye, Bo Jia

**Affiliations:** ^1^ Department of Oral Surgery, Stomatological Hospital, Southern Medical University, Guangzhou, China; ^2^ Department of stomatology, Guangdong Provincial Corps Hospital, Chinese People’s Armed Police Force, Guangzhou, China; ^3^ Center of Regenerative Medicine, Renmin Hospital of Wuhan University, Wuhan University, Wuhan, China; ^4^ School of Stomatology and Medicine, Foshan University, Foshan, China

**Keywords:** organoids, disease model, vascularization, immune environment, extracellular matrix

## Abstract

Traditional cell lines and xenograft models have been widely recognized and used in research. As a new research model, organoids have made significant progress and development in the past 10 years. Compared with traditional models, organoids have more advantages and have been applied in cancer research, genetic diseases, infectious diseases, and regenerative medicine. This review presented the advantages and disadvantages of organoids in physiological development, pathological mechanism, drug screening, and organ transplantation. Further, this review summarized the current situation of vascularization, immune microenvironment, and hydrogel, which are the main influencing factors of organoids, and pointed out the future directions of development.

## Introduction

Organoids are a three-dimensional (3D) cell culture derived from pluripotent stem cells [PSCs, including embryonic stem cells (ESCs) and induced pluripotent stem cells (iPSCs), tissue stem cells, adult stem cells (ASCs), or tumor stem cells] *in vitro*. Organoids reproduce the morphological structure, physiological function, gene specificity, and other characteristics of the source tissue. At present, the word “organoid” refers to a 3D culture described publicly for the first time by Sato et al. (2009). Under certain cytokine-rich matrix culture conditions, single LGR5+ intestinal stem cells (ISCs) can form and maintain an organ-like structure composed of different cells with a crypt-villus structure, which is highly similar to that of freshly isolated small intestinal crypt-villus structure (Sato et al., 2009). Thereafter, organoids were developed and used as a new research model independent of traditional cell lines and heterogeneous animal models.

According to the PubMed literature search by Simian et al., the number of publications that used “organoids” as keywords has increased sharply since 2011, which reveals the vigorous development of the research on organoid models (Simian and Bissell, 2017). Organoids provide a completely different new research model for medical research, including histopathology (Pott et al., 2018), drug development and screening (Fong et al., 2018), and precision medicine (Pauli et al., 2017). However, compared with the traditional cell line model and xenograft animal model used for decades in research and practice, many researchers still know little about organoids, and the model is still in its infancy.

Herein, we will systematically show, summarize, and review the advantages and disadvantages of disease models; integrate the research of organoids in cancer, infectious diseases, genetic diseases, regenerative medicine in recent years; and further discuss the main limitations and breakthroughs. These will help more readers understand the application of organoids, improve disease research, and promote organoid development.

### Comparison of Organoid Models With Other Models

Human tissue is a precise and rigorous whole composed of various cells, a specific structure, and its surrounding microenvironment. The cell line model and xenograft animal model are the most commonly used research models, which provide a platform for the development of medicine. However, the complexity of physiological and pathological responses between a single cell line and humans with multiple cells is unparalleled. There is species specificity between the physiological and pathological responses of humans and other animals. However, their limitations cannot meet the needs of all research kinds. Thus, there is an urgent need for a model that conforms to the human physiological and pathological environment as much as possible for scientific research (Table 1).

**TABLE 1:** Advantages and limitations of cell lines and xenografts.

Models	Advantages	Limitations
Cell lines	Low cost and save time	Only represent a subgroup of research objects
	Easy to maintain and expand	Loss of the original tissue heterogeneity
	High-throughput drug screening	Cannot receive signals, such as those *in vivo*
	Suitable for gene editing	
Patient-derived xenograft animal models	Maintain the three-dimensional structure	More time-consuming and expensive
	Interact with the host matrix and immune cells	Animal cells replace the primitive human cells
	Better preclinical treatment strategies	Loss of the original tumor heterogeneity

### Cell Lines

In 1907, Harrison inoculated isolated frog embryo medulla tissue onto frog lymph blocks, thus opening up an experimental method of cultivating cells on glassware. This experimental method is the beginning of tissue culture technology, from which cell lines became inseparable from the development of medicine. Cell lines have remarkable advantages. For example, they are inexpensive, easy to maintain, and expand, and the technology is relatively simple. Cell lines are suitable for high-throughput drug screening, and multiple cell line populations with histological and genetic changes can simulate different clinical situations and drug responses (Iorio et al., 2016; Lee et al., 2018a). Cell lines are also suitable for gene editing (Tang et al., 2016; Liao et al., 2017; Xia et al., 2017). Through superior gene editing, significant progress was made in discovering carcinogenic and molecular targets *in vitro*. Therefore, cell lines are the most widely used research model in the past few decades, as they play an irreplaceable role in disease development research and drug screening.

However, the cell line model has two main limitations. The cell line consists of a single cell, likely representing a subgroup of research objects (Ben-David et al., 2018). First, cell line survival is affected by pressure that is highly sensitive to culture conditions, and persistent instability can quickly lead to the heterogeneity of the cell line. With time, the cell line finally loses the ability to generalize the heterogeneity of the original tissue. Second, the cell line model has two-dimensional (2D) growth, with neither external signals from adjacent cells nor distal external signals from the circulatory system. Therefore, this model cannot reflect vital structures and microenvironments that usually occur under specific physiological or pathological conditions. For example, in tumor development, microenvironmental factors such as blood and lymphatic system (Mazzone and Bergers, 2019), surrounding fibroblasts (Hussain et al., 2020), and mechanical stimulation (Sewell-Loftin et al., 2020) affect the behavior and molecular characteristics of cancer cells and thus change the invasive ability of cancer, resistance to chemotherapy, and growth *in vivo*. Given these main limitations, traditional cell line disease models often misestimate the disease development and treatment response *in vivo*, which seriously limits the application of cell line models in experimental research.

### Patient-Derived Xenografts

PDX are the most common tumor models established by transplanting human tumor tissues/cells in animal hosts (usually immunodeficient or humanized mice) (Lai et al., 2017). The first patient-derived tumor xenograft animal model was established in the 1950s (Toolan, 1953). In research using PDX model, the aim was to obtain a group of animals with diseases similar to those of humans and conduct medical experiments to obtain preclinical results (Byrne et al., 2017). PDX can grow in a 3D microenvironment rich in nutrients and oxygen, can interact and communicate with the host matrix and immune cells, and can maintain the original 3D structure of the tumors and original genomic and phenotypic characteristics. Therefore, they are more suitable for use in predicting drug responses (Gao et al., 2015; Drapkin et al., 2018) and in preclinical host trials (Farago et al., 2019; Savaikar et al., 2020) and for testing the efficacy of new strategies (Stewart et al., 2017). Based on the above advantages, PDX have excellent predictive value and are indispensable for result authentication in essential clinical experiments.

However, the PDX model still has some limitations. PDX models are more time-consuming and expensive than other research models. Moreover, experiments using PDX require ethical approval and consideration of animal welfare and are strictly regulated (Biller-Andorno et al., 2015). With tumor subcultures, mouse stromal cells will replace the primitive human stromal cells and immune cells (Braekeveldt et al., 2016). During the administration of PDX, PDX-specific copy number alterations gradually disappear and primitive heterogeneity was lost (Ben-David et al., 2017; Ben-David et al., 2019). The genomic map and tumor microenvironment will affect treatment response. In the era of targeted and precision therapy, clinical results are inconsistent with the results of animal experiments, which lower the predictive value of PDX.

### Organoids

Given the limitations of cell lines and PDX, scientists constantly aim to construct a new research model, which has the advantages of both cell lines and PDX. Until 2009, Sato et al. (2009) first described an organoid culture system that allows ISCs to expand *in vitro* for a long time in a 3D extracellular matrix (ECM), which opened the prelude to the new era. In the past 10 years, organoids are extensively applied to understand stem cell biology, organogenesis, and human pathology and made considerable breakthroughs in disease mechanism research, drug screening, and precision treatment.

Organoids have significant advantages, as they require relatively less time and money but offer a high success rate. Organoids retain the 3D structure of the original tissues, faithfully summarizing the genetic and phenotypic heterogeneity of the original tissue (Cristobal et al., 2017). Organoids can be a platform in screening drugs with high efficiency and sensitivity, guiding clinical precision treatment and personalized treatment (Pauli et al., 2017). Organoids are also suitable for gene editing. For example, combining the CRISPR–Cas9 system and organoids provides a new opportunity to study tumor gene mutation (Drost et al., 2017). Organoids can be transformed into other models (Jian et al., 2020), cooperate and construct a biological bank (biobank) (Kim et al., 2019; Zhang et al., 2021), to establish a comprehensive and accurate experimental platform. However, organoids still have limitations. Thus, the second half of this review will focus on the current predicament and future development.

**FIGURE 1:**
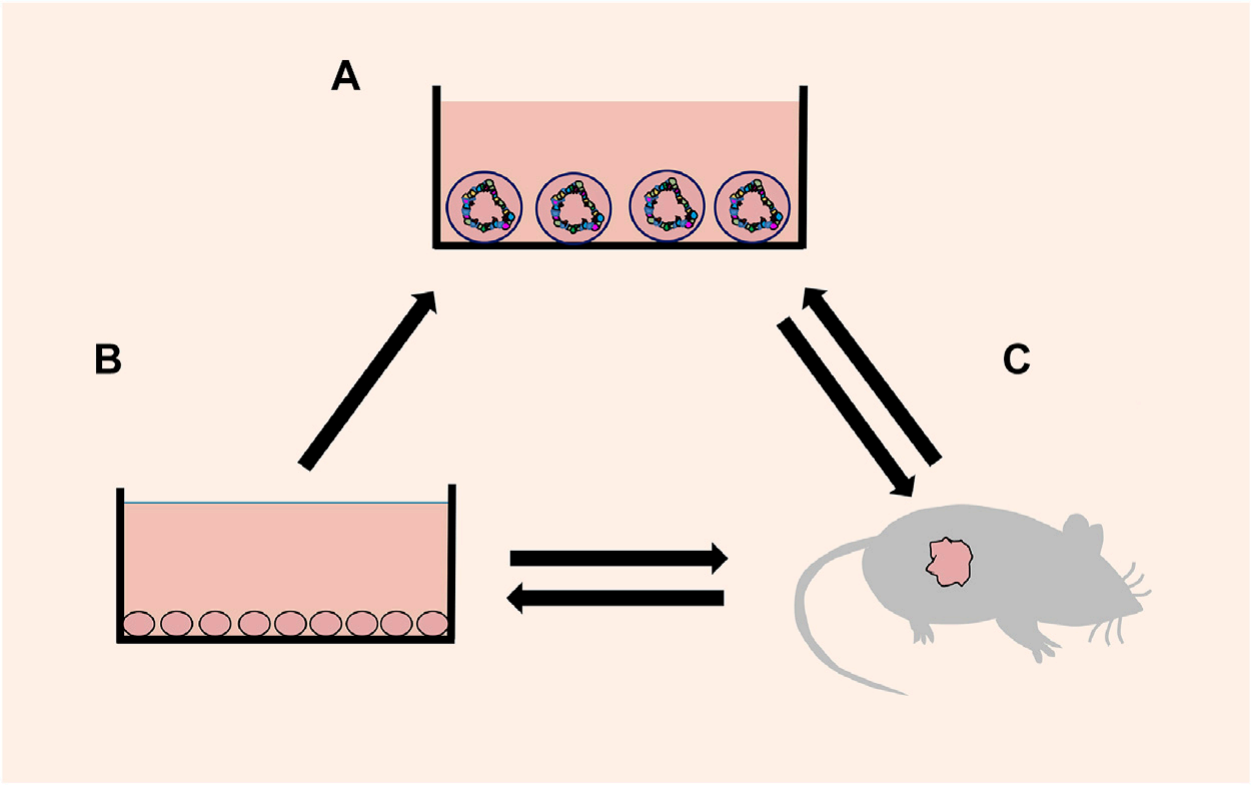
Schematic diagram of mutual transformation. **(A)** Organoids: a 3D cell culture, which reproduces the cell composition, morphological structure and other characteristics of the source tissue. **(B)** Cell lines: a 2D cell culture consisting of a single cell. **(C)** Xenografts: transplantation of human tissue/cells or directly induced animal models.

As a new research model and development platform, organoids fill the gap between the cell line and PDX (Figure 1), support the existing model system, and extend basic biological research, medical research, and drug discovery to the human environment related to physiology. In the future, the study of organoids has a broad prospect, and an increasing number of scientists will pay attention to organoids and combine them more widely and deeply with various fields of medical research.

In the past 10 years, organoids break through the simple physical contact between cells and form closer biological communication; i.e., organoids break through the dilemma that it is difficult for PDX to replicate physiological processes that exist only in humans. Moreover, we will take cancer, infectious diseases, genetic diseases, and regenerative medicine as examples to show the potential of organoids and attract more scholars to use this technology in the future.

## Organoids as Disease Models

### Organoids in Cancer Research

**FIGURE 2:**
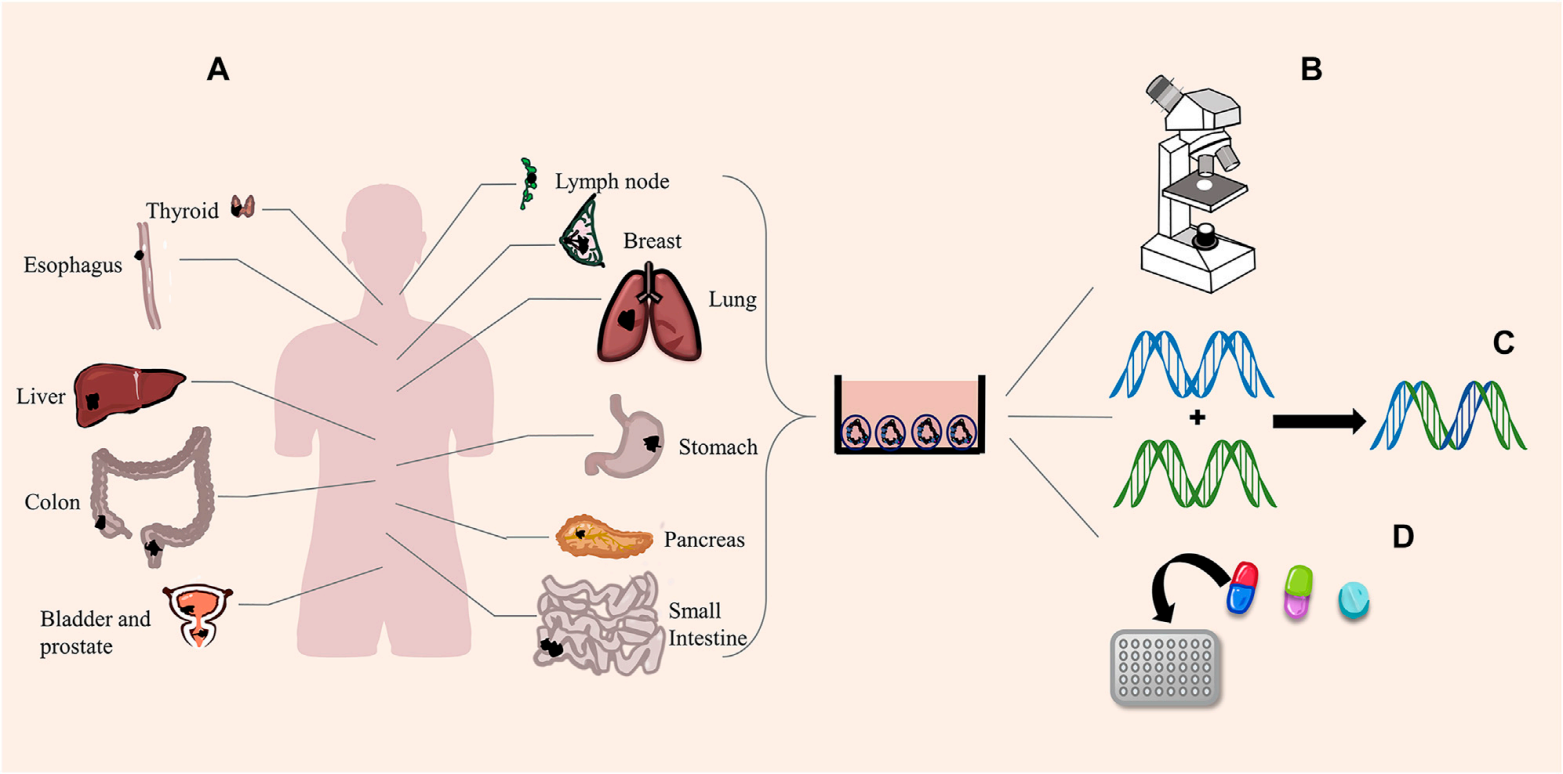
Tumor organoids and functions. **(A)** Scientists successfully cultured different cancer organoids. **(B)** Organoids perfectly simulate the pathological expression and tissue structure of tumors. **(C)** Organoids are suitable for gene editing. **(D)** High-throughput screening of drugs.

*Cancer* is a significant health problem worldwide. Scientists predict that the incidence of all cancer types will increase from 12 million in 2008 to 22 million by 2030 (Berkers et al., 2019). The incidence of and mortality from cancer increase in developing countries, so cancer research has a long way to go (Feng et al., 2019). We have performed much in-depth investigation on the origin, pathology, and genetics of cancer in the past few decades, and we found that the knowledge gained from cell lines and PDX is not entirely consistent with the actual clinical situation. We are increasingly aware that cancer has different heterogeneity and plasticity; thus, how to treat it accurately is very important to the survival and prognosis of patients with cancer. The recently developed 3D culture technology has led to the development of new and more physiological organoids, thus creating new and more targeted therapies. In the more than 10 years since the emergence of organoids, scientists successfully cultivated all kinds of tumor organoids, such as bladder cancer (Lee et al., 2018b), colorectal cancer (van de Wetering et al., 2015), lung cancer (Dijkstra et al., 2020), liver cancer (Nuciforo et al., 2018), prostate cancer (Puca et al., 2018), breast cancer (Han et al., 2020), gastric cancer (Yan et al., 2018), pancreatic cancer (Seino et al., 2018), lymphatic cancer (Tian et al., 2015), esophageal cancer (Li et al., 2018), thyroid cancer (Sondorp et al., 2020), among others (Figure 2). These studies have shown that tumor organoids are highly similar to the original tumors in terms of genetic specificity, epigenetic characteristics, morphology, metabolism, proliferation rate, and metastatic potential. Moreover, organoids build a more suitable platform for exploring new therapeutic targets and high-throughput screening of drugs.

For example, in lung cancer, the global estimates of cancer morbidity and mortality compiled by the International Agency for Research on *Cancer* in 2018 show that lung cancer is the most common cancer (accounting for 11.6% of total cases) and the leading cause of cancer death (18.4% of total cancer deaths). There are 2.1 million new cases and 1.8 million deaths annually (Bray et al., 2018). The emergence of organoids brings new impetus to the research of lung cancer.

Lung cancer has various manifestations, and whether organoids can reproduce the unique tissue structure and epigenetics of patients is the first problem to address. In one study, the structure of the organoids of patients with lung cancer ranged from thin-walled cystic structures to dense spherical cell clusters, containing 50% mutations, and more than 70% of the cells expressed acetaldehyde dehydrogenase, which maintained the biological characteristics of tumor biopsies (Jung et al., 2019). Once the primary lung cancer tissue is obtained, organoids can be continuously regenerated and replicated *in vitro*, and the cytological characteristics of the tumor remain stable. The clinical markers remained unchanged during the passage, which lays a foundation for follow-up research. For lung cancer types in which patient samples are lacking, gene editing is combined with organoids to create new possibilities. Some scholars used the CRISPR–Cas9 system to edit multiple genes in lung organoids and showed that the transfected organoids expressed primary cell-specific markers of lung squamous cell carcinoma, such as cytokeratin 5, p63, and epithelial cell adhesion molecule. This kind of gene-edited organoids can be applied in immune checkpoint therapy combined with drug-screening platforms (Hai et al., 2020). Gene editing is a feasible method for rapid mass production of organoids, and this significantly facilitates lung cancer research. However, gene editing can lead to mutations, instability, and uncertainty. Thus, whether the tumor organoids obtained by gene editing are entirely consistent with the source patients is a question worth considering. Drug screening is a strict demonstration process, and the effect of drugs screened through organoids needs to be verified by more experiments. Moreover, this process provides us with an economical and rapid method of studying the critical targets of the tumor.

Drug development and screening is a rigorous and essential process. In the past, PDX have been used as the last barrier before clinical trials. However, the complexity and high cost of PDX limit large-scale drug trials. A current comparative study of non-small cell lung cancer showed that the sensitivity of KRAS-mutant organoids to the MEK inhibitor trametinib was consistent with the relative PDX model. By contrast, organoids without KRAS mutations were as resistant as relative PDX (Shi et al., 2020a). This finding suggests the feasibility of replacing PDX with organoids as a drug testing platform, which significantly reduces the cost of large-scale screening. As an *in vitro* model, organoids still lack many mechanisms that affect drug responses, such as drug metabolism and tissue infiltration; thus, confirming the consistency and effectiveness of the organoids’ response to drugs *in vivo* and *in vitro* is important. Wang et al. established a 15 patient-derived non-small cell lung cancer organoid model with human epidermal growth factor receptor 2 mutation. Drug screening for those organoids shows that pirotinib had a more significant inhibitory effect on organoid proliferation than afatinib *in vitro*. In the final clinical trial, the results were the same as drug screening of organoids, and pirotinib showed good efficacy (Wang et al., 2019). This study shows that organoids are more feasible in clinical tumor treatment and can help shorten the absolute distance between clinic and laboratory. Considering that lung cancer has various biological and molecular subtypes, genetic heterogeneity is an essential factor that affects the response to drug therapy. We still need to establish biobanks with a large number of data. The era of single-drug treatment for all patients has passed, and the future focuses on the era of personalized and accurate treatment. Organoids have a great application prospect in biobanks. Kim et al. established a biobank derived from 80 lung cancer organoids. The organoid biobank covers more than 95% of lung cancer types, including adenocarcinoma, squamous cell carcinoma, small cell carcinoma, adenosquamous cell carcinoma, and large cell carcinoma. They screened targeted drugs such as docetaxel, olaparib, erlotinib, and crizotinib, showing that organoid biobanks can predict patient-specific drug responses *in vitro* as well as proof-of-concept studies of new targeted drugs based on genetic changes (Kim et al., 2019). The development of organoid models is significantly conducive to drug development and screening. In the future, we also need to increase the sample size of organoid biobank. Through multi-omics technology to analyze tumor organoids, coupled with large-scale drug screening, data are analyzed by artificial intelligence. Finally, the multi-omics data of tumor organoids are related to drug sensitivity and realize personalized and accurate treatment.

By understanding the biology of tumor organoids and apply them in clinical practice, a preclinical cancer model is needed. We have discussed the main characteristics and application of organoid technology in cancer. Although it is still in the early stage of exploration, the prospect is up and coming. The results of this series of organoid studies on various cancers continue to send us a clear message: Tumor organoids can maintain the phenotype and genetic heterogeneity of the primary tumor. Tumor organoid research can deepen our understanding of the mechanism of tumorigenesis and provide a possible method of treating tumors. Moreover, organoids can carry out high-throughput drug screening and contribute to the development of precise and personalized treatment.

### Organoids in Hereditary Diseases

Approximately 5.3% of infants will suffer from genetic diseases when followed up until age 25 years (Verma and Puri, 2015). Many genetic diseases take time to develop in a specific tissue microenvironment, and a traditional single cell line cannot simulate these diseases. In addition, xenograft models do not entirely simulate the characteristics of genetic diseases in humans, such as diseases that affect neurodevelopment and the brain. Thus, further research on cell lines and xenografts in genetic diseases is not needed. Organoids can summarize the organ’s structure and function and retain the tissue’s original genetic and epigenetic state, which are suitable for gene editing. These aspects are crucial for modeling genetic diseases caused by one (single gene) or multiple (multifactor) abnormalities.

Genetic diseases are mainly caused by mutations of various genes, which make it difficult for cell lines and xenografts to simulate these genetic diseases. Thus, the best choice is to use organoids from the source patient (Table 2). Cystic fibrosis (CF) is an autosomal recessive hereditary disease. The imbalance of chloride channels controlled by transmembrane conductor receptors affects the epithelial cells of many organs such as the lung, pancreas, sweat gland, liver, kidney, and intestine. The corresponding imbalance results in pancreatic insufficiency, gastrointestinal symptoms, and progressive lung diseases. In the past, symptomatic treatments, such as mucolytic agents, anti-inflammatory drugs, and antibiotics, were employed (Villanueva et al., 2017). Because organoids perfectly maintain the genetic specificity of the source tissue, the research on organoid technology to unravel the complex molecular mechanisms and drug screening has increased. Herein, we focused on the breakthrough in CF owing to the combination of organoids and gene-editing technology. The combination of organoids and gene-editing technology brings new hope for the complete cure of CF. In 2013, Schwank et al. used the CRISPR–Cas9 genome-editing system to correct the site of the CF transmembrane conductor receptor (CFTR) through homologous recombination in CF patient-derived intestinal and colonic organoids, and this provided a theoretical basis for gene correction by homologous recombination in primary ASCs from patients with single-gene hereditary defects (Schwank et al., 2013). Subsequently, gene editing corrected proximal lung organoids and patient-derived adenocarcinoma cell line CFPAC-1 (Ruan et al., 2019), intestinal organoids, and airway epithelial cells from patients with CF (Maule et al., 2019), and nasal mucosa of CF mice and patient-derived small intestinal organoids (Dekkers et al., 2013; Vidović et al., 2016). The chloride channel has been effectively repaired and specific function restored, which shows the feasibility of implanting the edited organoids back into the body. In a recent study, Maarten et al. described a biobank of CF intestinal organoids, representing 664 patients, and believed that 20% of them could theoretically enable efficient repair of nonsense mutations in CFTR through adenine base editors (Geurts et al., 2020). Although gene-edited organoids have made some achievements in animal models, their efficiency is very low, and the success rate is not high. More importantly, the safety, long-term stability, and functionality of gene-edited organoids should be tested over time.

**TABLE 2:** Unique advantages of organoids in hereditary disease.

Report year	Cellular input	Organoids	Disease	Applications
**2013**	Murine and human intestinal stem cells	Epithelial organoids	Cystic fibrosis	Disease model; CRISPR-based genome editing; Gene therapy Schwank et al. (2013)
**2015**	Human rectal stem cell	Intestinal organoids	Cystic fibrosis	Disease model; Gene therapy Vidović et al. (2016)
**2016**	Human iPSC	Optic cup organoids	Leber congenital amaurosis	Disease model; Pathological mechanism; Drug screening Parfitt et al. (2016)
**2017**	Mouse Liver tissues	Liver organoids	Alagille syndrome	Verify the characteristics of the Jag1Ndr/Ndr mouse model Andersson et al. (2018)
**2017**	Human iPSC	Forebrain-type organoids	Miller-Dieker syndrome	Disease model; Pathological mechanism; Modeling of cell-cell communication Iefremova et al. (2017)
**2017**	Human iPSC	Cerebral Organoids	Miller-Dieker syndrome	Disease model; Pathological mechanism; Single-cell RNA sequencing Bershteyn et al. (2017)
**2017**	Human iPSC	Hepatic organoids	Alagille syndrome	Disease model; CRISPR-based genome editing; Gene therapy Guan et al. (2017)
**2018**	Human iPSC	Cerebral organoids	Sandhoff disease	Disease model; Pathological mechanism Allende et al. (2018)
**2018**	Human iPSC	Retinal organoids	Retinitis pigmentosa	Disease model; CRISPR-based genome editing; gene therapy Deng et al. (2018)
**2018**	Human iPSCs	Retinal organoids	Leber congenital amaurosis type 10	Drug screening Dulla et al. (2018)
**2019**	Human iPSCs	Cerebral organoids	Juvenile form of neuronal ceroid lipofuscinosis	Disease model and pathological mechanism Gomez-Giro et al. (2019)
**2019**	Human iPSCs	Proximal lung organoids	Cystic fibrosis	Disease model, CRISPR-based genome editing, and gene therapy Ruan et al. (2019)
**2019**	Human primary rectal stem cells	Intestinal organoids	Cystic fibrosis	Disease model, CRISPR-based genome editing, and gene therapy Maule et al. (2019)
**2020**	Human iPSCs	Cerebral organoids	Schizophrenia	Disease model and pathological mechanism Benson et al. (2020)
**2020**	Human iPSCs	Retinal organoids	X-linked retinitis pigmentosa	Disease model, pathological mechanism, and CRISPR-based genome editing Lane et al. (2020)
**2020**	Human intestinal stem cells	Intestinal organoids and airway organoids	Cystic fibrosis	Organoid biobank, adenine-based editing gene therapy Geurts et al. (2020)

iPSCs, induced pluripotent stem cell.

With the establishment and application of organoids, this 3D method has been developed rapidly in basic research and transformation research of genetic diseases. Organoids and gene editing show the potential to completely cure CF, and CF may 1 day be cured by this technique. Organoids create an exciting new opportunity for human genetic diseases *in vitro* and bring the dawn for the final cure of genetic diseases through gene-editing technology.

### Organoids in Infectious Diseases

**FIGURE 3:**
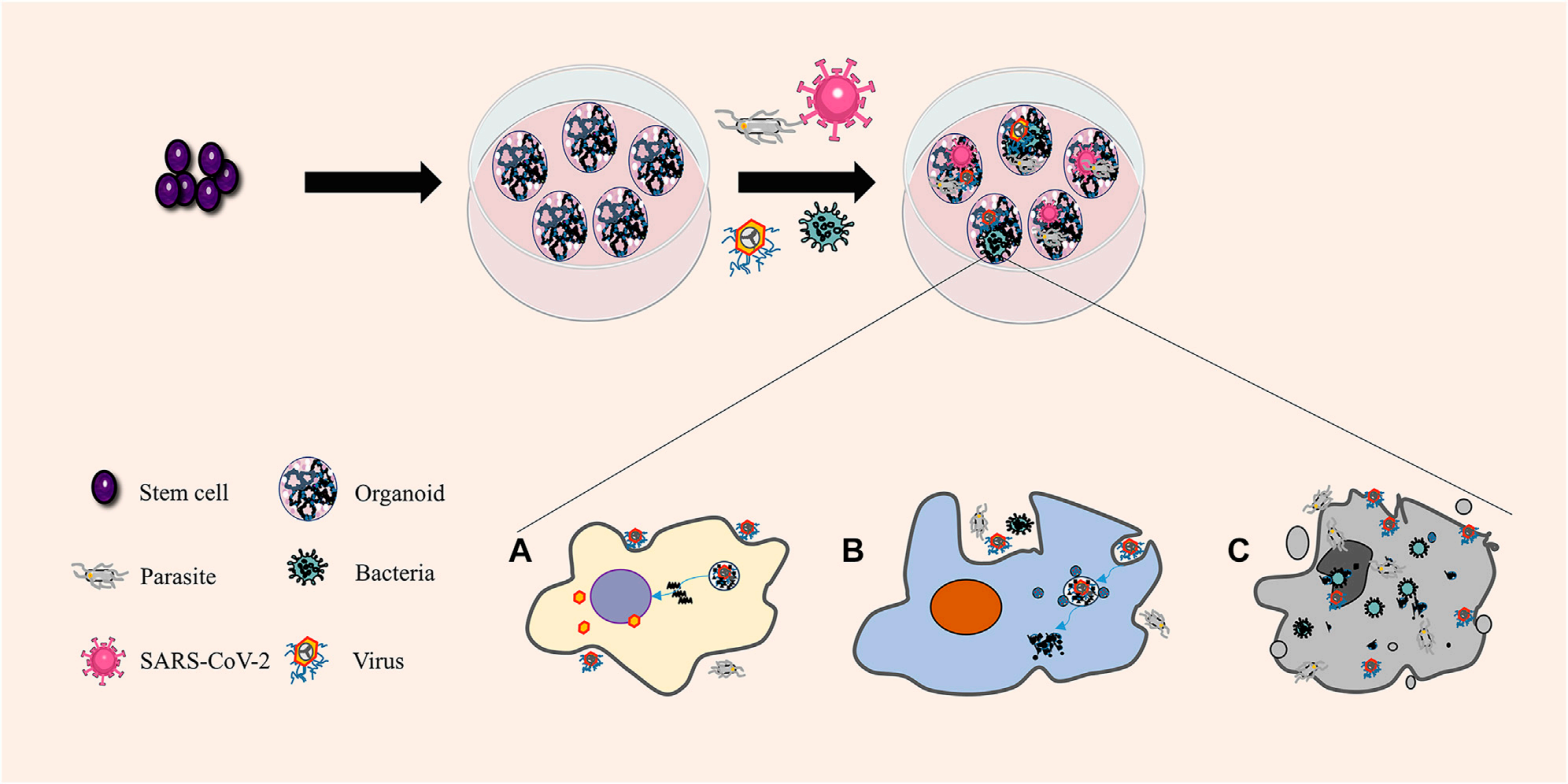
Organoids in infectious diseases. Organoids are unique and composed of various differentiated cells. Different cells can connect with the pathogens simultaneously and react differently in the same organoids, consistent with the response of the human body. **(A)** Pathogenic microorganisms use the host to reproduce. **(B)** The pathogenic microorganism is killed and decomposed by the host. **(C)** Host cell lysis and death.

Research on human infectious diseases is limited by the lack of normal human physiological and pathophysiological functional models. In the traditional research model, the classical single-cell culture system cannot reproduce complex and dynamic responses or cell-cell interactions. Xenograft animal models may respond similarly to human models, but species-specific differences may lead to an unfaithful display of disease cycles and human responses. The application of organoids allows for the examination of complex physiological or pathological processes in more similar structures under *in vivo* conditions and paves the way for studying infection and host–source interaction (Figure 3). A model of the interaction among parasites, bacteria, viruses, and other pathogens and their hosts has been established, such as cryptosporidium and small intestine and lung organoids (Heo et al., 2018), Zika virus and brain organoids (Cugola et al., 2016), hepatitis B virus and functional liver organoids (Nie et al., 2018), and *Salmonella typhimurium* and intestinal organoids (Forbester et al., 2018).

Regarding severe acute respiratory syndrome coronavirus 2 (SARS-CoV-2), as of July 2021, nearly 180 million cumulative cases and more than 4 million deaths were confirmed worldwide. Common symptoms of coronavirus disease 2019 (COVID-19) include fever, cough, and shortness of breath, and the most common cause of death is respiratory failure, combined with metabolic complications in other body systems, including the cardiac, renal, nervous, and gastrointestinal systems (Wiersinga et al., 2020). Patient-derived organoids have various cell types, molecular expression patterns, genetic markers, etc., contained in the corresponding body organs, which can simulate the pathogenic process of human infection, reflect the whole process of infectious diseases intuitively, and provide the possibility for studying the characteristics of pathogens and the interaction between human hosts and pathogens. With the recent SARS-CoV-2 pandemic, determining appropriate therapeutic drugs is the top priority. Han et al. developed human pluripotent stem cells lung organoids (hPSC-LOs) for SARS-CoV-2 and SARS-CoV-2 colon organoids (hPSC-COs), and high-throughput screening showed that imatinib, mycophenolic acid, and quinacrine dihydrochloride significantly inhibited SARS-CoV-2 (Han et al., 2021). This indicates the use of SARS-CoV-2-infected hPSC-LOs and hPSC-COs as valuable research models for screening drugs to identify candidate therapeutic agents for COVID-19. In addition, scientists have established models of common complications in patients with SARS-CoV-2 infection, for example, the SARS-CoV-2 infection of a human intestinal organoid model (Lamers et al., 2020; Stanifer et al., 2020; Zhou et al., 2020), SARS-CoV-2 infection of a human renal organoid model (Monteil et al., 2020; Xia et al., 2020), SARS-CoV-2 infection of a human brain organoid model (Jacob et al., 2020; Ramani et al., 2020), and SARS-CoV-2 infection of a human liver organoid model (Schultz et al., 2020; Yang et al., 2020; Zhao et al., 2020). These models laid the foundation for the organoid biobanks of SARS-CoV-2 infection, which provide valuable models for drug screening and evaluation of antiviral therapy. With the use of increasingly different SARS-CoV-2 infection organoids, we will know more about the characteristics and disease mechanism of SARS-CoV-2 from different aspects.

In addition to existing drug screening, novel drug development and drug combinations are top priorities of research. SARS-CoV-2 uses angiotensin-converting enzyme 2 (ACE2) and transmembrane serine protease 2 as the main entry point into host cells (Ziegler et al., 2020), and as confirmed in related organoids (Mahalingam et al., 2021), many studies on drug developments have focused on these two targets. Some scientists have designed a humanized decoy antibody (ACE2-Fc fusion protein) that neutralizes SARS-CoV-2 infection, transforming angiotensin II (Ang II) into Ang II–VII and activating natural killer cells (Huang et al., 2021). This antibody may be applied in health care workers or high-risk groups to prevent SARSCoV-2 infection. In the combination drug therapy, anti-androgen drug therapy can reduce the expression of ACE2 and protect hESC-derived lung organoids from SARS-CoV-2 infection (Samuel et al., 2020). Combining human-recombinant soluble ACE2 or human neutralizing antibody with remdesivir effectively inhibits SARS-CoV-2 replication and significantly improves efficacy (Pei et al., 2020; Monteil et al., 2021). Because of the excellent characteristics of organoid models, the drugs and treatment methods developed using SARS-CoV-2 infection organoids are believed to be more reliable and effective, so clinical trials are conducted more quickly.

Organoids are different from cell lines and xenograft animal models and ideally represent all the cellular components of organs of specific species. Theoretically, they are very suitable for investigating infectious diseases, especially those caused by pathogens. Considering the toxicology of drugs, it will be more reasonable to transplant organoids into animal models and then verify the efficacy of related drugs. Organoids bring about a new method of drug screening, treatment, and vaccine of COVID-19, which will help contain the pandemic.

### Organoids in Regenerative Medicine

Regenerative medicine applies biological and engineering theories and methods to restore lost or functionally damaged tissues and organs. Regenerative medicine restores the function of tissues or organs in patients with severe injury or chronic diseases. Tissue regeneration and repair face three main problems, namely, donor shortage (Dwyer et al., 2021), immune rejection (Loupy and Lefaucheur, 2018), and ethical issues (Lieber et al., 2018), promoting the search for alternatives. Organoids can be derived from the patient’s PSCs and ASCs. They rapidly reproduce *in vitro* and retain the 3D structure, function, heredity, and phenotypic specificity of the original tissues. These advantages are essential for transplantable, immune, and functional tissues or organs *in vitro*, which undoubtedly brings a new dawn for regenerative medicine (Table 3).

**TABLE 3:** Organoids used regenerative medicine research.

Report year	Cellular input	Organoids	Transplant site	Results
2014	Mouse small intestinal stem cells	Small intestinal epithelial organoids	Mouse colon	Reconstitute self-renewing epithelial and show unique features of the small intestines Fukuda et al. (2014)
2016	Human primary bronchial epithelial cells + peripheral cells	Airway organoids	Kidney capsule of NSG mice	Self-organize and mature toward lung tissue-like structures; remarkable epithelial plasticity Tan et al. (2017)
2017	Mouse- and human-induced fetal intestinal progenitor cells	Spherical and budding intestinal organoids	Injured colonic tissues	Reconstitute colonic and intestinal epithelia Miura and Suzuki (2017)
2017	Human primary cholangiocyte	Extrahepatic cholangiocyte organoids	Surgical defect in mouse gallbladder wall	Colonize physiological niche and regenerate part of the biliary tree Sampaziotis et al. (2017)
2017	Mouse newborn dermal cells and adult epidermal cells	Skin organoids	Mouse full-thickness skin wound	Well-formed reconstituted skin with robust hair growth; cyclic regeneration Lei et al. (2017)
2017	Human pluripotent stem cells	Intestinal organoids	Mouse injured intestinal mucosa	Organoid survival, engraftment, and wound repair Cruz-Acuña et al. (2017)
2018	Human and primary murine hepatocytes	Hepatocyte organoids	Mouse damaged mouse liver	Recapitulate the proliferative damage-response and demonstrate significant graft expansion Hu et al. (2018)
2018	Human colonic stem cell	Colon organoids	Mouse colon	Reconstruction of the human colon epithelium and multi-differentiation capacity Sugimoto et al. (2018)
2019	Human islet cells and amniotic epithelial cells	Islet organoids	Kidney capsule of diabetic mice	Viable and functional islet organoids; diabetes reversal Lebreton et al. (2019)
2019	Mouse sweat gland epithelial cells	Sweat gland organoids	Mouse back wound and claw pad with sweat gland injury	Regeneration of *epidermis* and sweat glands Diao et al. (2019)
2019	Human mesenteric lymph nodes and primary stromal progenitors	Synthetic lymphoid organoids	Site of resected mouse lymph nodes	Restore lymphatic drainage and perfusion and support the activation of antigen-specific immune responses Lenti et al. (2019)

As a valuable tool for the research and treatment of intestinal diseases, intestinal organoids are the most promising direction in organoid regenerative medicine research. In the beginning, small intestinal organoids derived from stem cells were transplanted into the mouse intestinal model, and epithelial crypt-like structures, villi, and Paneth cells were found, which had the characteristics of the primitive intestines (Fordham et al., 2013; Fukuda et al., 2014). That study points to the future opportunities for specific gastrointestinal regeneration in patients, and scientists made various efforts in this direction. Others found that intestinal organoids derived from genetically programmed cells had ISC properties. Organoids transplanted into the damaged colon resembled morphologically and functionally at least 3 months (Miura and Suzuki, 2017). Furthermore, Sugimoto et al. established an orthotopic xenograft system for normal human colonic organoids, enabling the stable reconstruction of human colonic epithelium *in vivo* for more than 10 months (Sugimoto et al., 2018). Organoid transplantation is a rigorous technique that needs time testing. However, whether the implanted organoids can be thoroughly combined with the primary tissues and maintain long-term functional stability still needs further investigation. All the aforementioned studies have shown the importance of organoids in regenerative medicine. However, the application of organoids in human transplantation still requires a suitable stent, which is necessary to reduce tissue rejection and incompatibility. Scientists designed synthetic hydrogels with a four-armed, maleimide-terminated poly (ethylene glycol) (PEG) macromer starting with the carrier (Cruz-Acuña et al., 2017). Synthetic hydrogels support the growth and expansion of human intestinal organoids *in vitro* and can be used as injection carriers for transport to the damaged intestinal mucosa to promote the healing of an intestinal injury. Organoid transplantation carriers should have the following characteristics: biocompatibility, nontoxicity to the human body and organoids, suitable viscosity, easy to use, and remain in the tissue defect for a long time. In addition, the structure facilitates the connection between organoids and primary tissues.

Owing to the cultivation of various kinds of patient-derived organoids *in vitro*, we can skillfully avoid or solve donor shortage, immune rejection, and ethical problems in regenerative medicine. At present, the size and proportion of organoids are still a problem in regenerative medicine. Most organoids are still in micron size and have no vascular system and peripheral immune microenvironment; thus, a large-scale reorganization experiment on organoids is required. Moreover, whether an organoid can be combined with the target tissue and form a standard functional unit is the criterion for successful implantation. However, whether the proportion of all cell kinds in organoids is consistent with that of the target tissue and whether it can be maintained during proliferation and differentiation are questions to consider. In the future, continuous development and improvement of organoids will create conditions for organ expansion *in vitro*, functional transplantation into patients, and reconstruction of tissue defects.

Organoids can be cultured by ESCs (Moris et al., 2020), iPSCs (Xie et al., 2020), ASCs (Huch et al., 2015), or tumor stem cells (Shimokawa et al., 2017) under suitable culture conditions *in vitro*. The cultivation of different organoids requires corresponding cytokines and culture matrices to form a specific microenvironment. The most commonly used culture conditions of organoids have two parts: 1) hydrogels, which are a 3D scaffold for organoid culture that promote the aggregation of 3D-cultured cells and produce the polarity of cell arrangement, and (Simian and Bissell, 2017) cell growth regulatory components, which simulate the ecological conditions of organs in the body, providing various information to maintain the ability of stem cells to self-renew, proliferate, and differentiate.

The gold standard for cultivating organoids is to simulate the tissue microenvironment in the body as much as possible. Compared with the dynamic microenvironment *in vivo*, the aforementioned conventional culture conditions of organoids are insufficient, and many key factors are ignored. Organoids have 3 main limitations. First, Current organoids are relatively simple and do have a vascular system. *In vivo*, tissues are penetrated and complex vascular networks interact to allow the exchange of oxygen, nutrients, waste, and different biochemical interactions between tissues. By contrast, *in vitro*, the microenvironment of organoids is still incomplete to a large extent. Second, current organoids do not include two critical components of immune factors and cells. Organoids cannot completely simulate the immune microenvironment of various tissues or diseases and lack the physiological process of mutual communication between the organoids and the surrounding immune environment, so they cannot completely simulate the complete response to the corresponding drugs or other treatments. Third, the culture of organoids is inseparable from the matrix, and the composition, structure, chemical, and physical characteristics of the matrix will affect the composition of organoids. However, no matrix meets the needs of this new *in vitro* model, which significantly limits the large-scale promotion and use of organoids. In the following section, we will focus on these shortcomings faced by similar organs, show the efforts made by scholars to break through the shackles, and point to future directions of research.

## Challenges and Breakthroughs

### Organoids Vascularization

At present, organoids are still of micron size, and a particular gap still exists between their functions and normal tissues. A significant factor is the lack of a vascular system. When organoids grow to a certain extent, the cells in the center cannot get enough nutrition, and excretion of metabolic waste in the cells is difficult, limiting the popularization and application of organoids. Two main methods can be employed to construct vascularized organoids: one is “*in vivo* vascularization” by transplanting organoids into animal models, and the other is “*in vitro* vascularization,” which is implemented by combining gene editing, mixed cell culture, and microfluidic platform.

**FIGURE 4:**
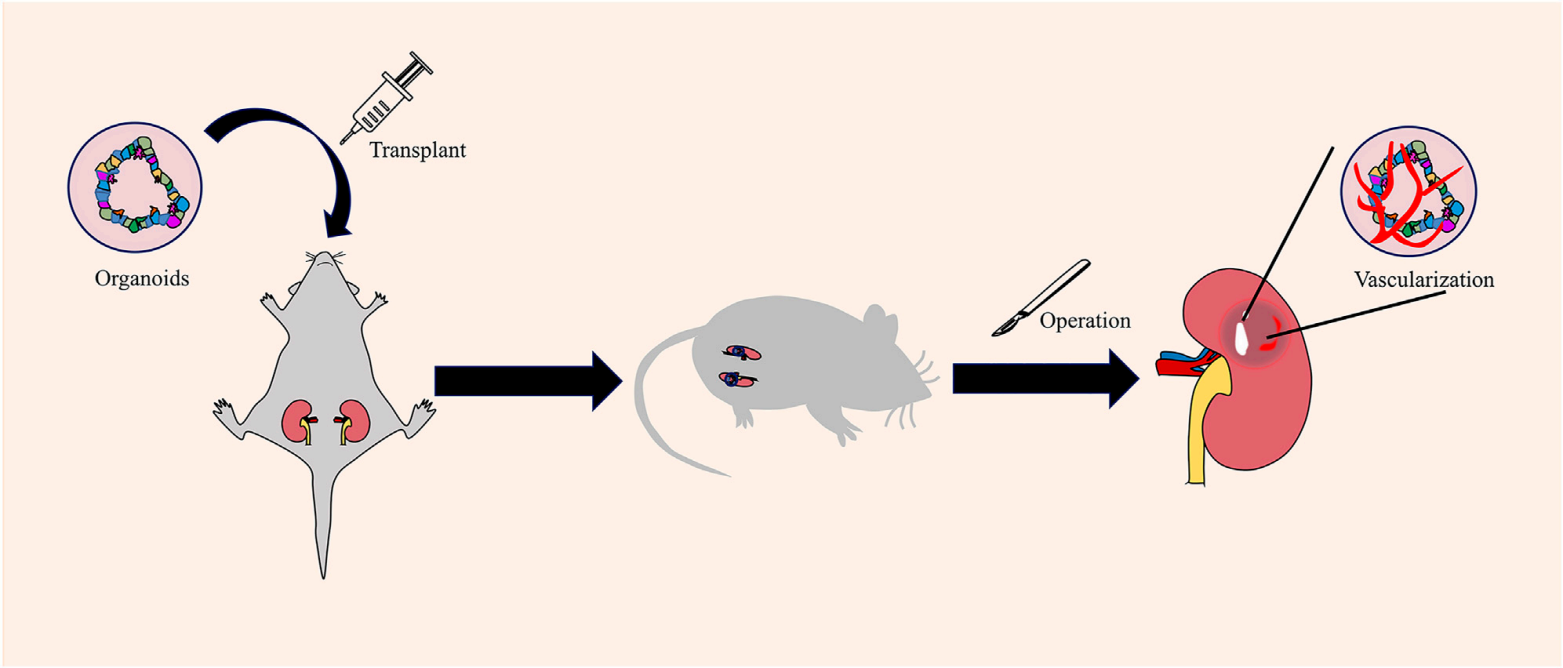
Obtaining vascularized renal organoids by *in vivo* vascularization. *In vivo* vascularization is carried out by transplanting organoids into the mouse. The capillaries around the mice will invade the organoid mass and establish vascular circulation.

In *in vivo* vascularization, vascularized organoids are constructed by transplanting the organoids into the corresponding animal model to promote the connection between the host animal model and the transplanted organoid model through blood vessels. The kidney is one of the organs closest to human blood vessels. The total amount of plasma filtered by humans every day is as high as 200 L. In humans, wastes are removed through the kidneys to maintain the balance of water, acid–base, and electrolytes in the body. Human-derived kidney organoids can be used as disease model; however, a key question is whether a functional vascular system can be constructed. Van et al. found that renal organoids gradually formed a vascular system by transplanting hPSC-derived renal organoids into the renal capsule of immunodeficient mice, and microscopic examination confirmed a functional vascular network between the subcapsular organoids and host blood vessels (van den Berg et al., 2018) (Figure 4). In a follow-up study, after vascularization in the host, renal organoids showed glucan and albumin size selectivity on the glomerular filtration barrier and proximal tubular dextran reuptake (van den Berg et al., 2020). Organoids with functional blood vessels are closer to the actual condition of human tissues in terms of structure and function, so they can be more suitable for establishing disease models and for clinical applications in regenerative medicine. Similarly, organoids are transplanted into the abdominal cavity and brain of mice to establish blood circulation (Raikwar et al., 2015; Mansour et al., 2018; Soltanian et al., 2019).

In in vivo vascularization, organoids are simple to use and a vascular system can be constructed when combined with an animal model. However, the close relationship between the organoids and xenografts. The internal environment makes the organoids form a vascular system with perfusion ability and form a staggered whole with the surrounding tissue so that organoids are really alive. By contrast, this *in vivo* vascularization model still cannot eliminate the limitations of traditional xenograft animal models. Even more worrying is that when human organoids are transferred into an animal, the complex vascular system will continue to reconstruct organoids. Thus, whether animals will gradually replace the cells, matrix, and structure of organoids needs more research.

*In vitro* vascularization means promoting the vascularization of organoids *in vitro* through the mixed culture of different cells, genetic engineering, or construction of various physical and chemical environments without other animal models (Table 4). For example, in brain organoids, some scientists use genetic engineering to make human cortical organs express EVT2 (the known regulatory gene of angiogenesis) to obtain vascular organoids having the characteristics of the blood–brain barrier (Cakir et al., 2019). Some scientists co-culture human ESCs or human multifunctional cells with human umbilical vein endothelial cells *in vitro* to produce organoids with a well-developed reticular or tubular vascular system (Shi et al., 2020b). The co-culture method depends on the introduction of other external angioblasts, which are simple but difficult to control. By contrast, the gene-editing method relies on the overexpression of some cytokines and inevitably faces some disadvantages, such as low success rate, gene mutation, technical difficulty, and high cost. Therefore, obtaining organoids with vascular characteristics through gene editing and co-culture is considered sufficient, but these organoids only deliver some oxygen and nutrients and are quite different from actual perfusion vessels. These organoids still need to be transplanted into animals to make the blood vessels genuinely functional.

**TABLE 4:** Vascularization of organoids *in vitro*.

Category	Report year	Methods	Organoids	Results
Co-culture	2018	Re-embed with endothelial cells	Brain organoids	Continuity of CD31-positive blood vessels inside the organoids Pham et al. (2018)
	2019	Co-culture with endogenous endothelial cells	Adipose tissue-like organoids	Connected to the recipient circulatory system Muller et al. (2019)
	2019	Vascular endothelial growth factor induction	Cerebral organoids	Vessel-like structures and blood–brain barrier characteristics Ham et al. (2020)
	2020	Co-culture with human umbilical vein endothelial cells	Cortical organoids	A well-developed mesh-like or tube-like vascular system Shi et al. (2020b)
	2021	Fusion with microvascular fragments	Islet organoids	Interconnection with surrounding blood vessels and restoration of normoglycemia Nalbach et al. (2021)
	2021	Fusion with microvascular fragments	Adipocyte organoids	Fabricate functional, vascularized, adipose-like organoids Strobel et al. (2021)
Organoids-on-a-chip	2019	Novel microphysiological platform	Retinal organoids	Vasculature-like perfusion and mature photoreceptor segments Achberger et al. (2019)
	2019	Culture underflow on millifluidic chips	Kidney organoids	Vascular network and enhanced cellular polarity and adult gene expression Homan et al. (2019)
	2020	A microfluidic platform named IFlowPlate	Colon organoids	A self-assembled vascular network and innate immune function Rajasekar et al. (2020)
Gene editing	2019	Ectopically express human ETS variant 2	Brain organoids	Vasculature-like structures and blood–brain barrier characteristics Cakir et al. (2019)
	2021	Overexpression of PROX1 and ATF5	Liver organoids	Vascular morphogenesis and improves native liver functions Velazquez et al. (2021)

The traditional *in vitro* vascularization method is a 2D culture model that can only cultivate organoids in a pre-added medium under a single culture condition. The complex environment in the body, such as mechanical stress, variable concentrations of nutrient factors, and fluid circulation, is difficult to simulate. Organoids-on-a-chip is the most effective model to simulate human functions. Compared with the static control group, the vasculature of renal tubules and glomerulus was enriched, and maturity was enhanced during the development of renal organoids in the presence of blood flow (Homan et al., 2019). In other studies, retina-on-chip provides vascular-like perfusion and, for the first time, can recreate the interaction of mature photoreceptor segments with the retinal pigment epithelium *in vitro* and could replicate the pathological mechanism and drug reaction *in vivo* (Rajasekar et al., 2020). The flow of the culture medium is essential for the formation of blood vessels, and organoids-on-a-chip can achieve a higher level of self-assembly, genuinely restoring specific physiological functions, pathological patterns, and drug responses of the prototype organ. Furthermore, a microfluidic platform called IFlowPlate can be used to culture up to 128 independently perfused and vascularized colon organoids *in vitro*. Tissue extraction is allowed for downstream experimental analysis (Rajasekar et al., 2020). The study is an essential step for the large-scale promotion and use of vascularized organoids. The current microfluidic platform is crude and semi-adjustable but more compliant with the development direction of organoids. To simulate the internal environment as accurately as possible, factors such as shape, concentration of cytokine composition, and flow rate must be considered because they can affect the vascularization of organoids. The microfluidic platform needs to be combined with artificial intelligence. The future microfluidic platform includes but is not limited to the following features: sleek appearance; use of electronic mechanical timing and quantitative injection of new culture medium; adjustable flow rate and direction of liquids; adjustable temperature, humidity, oxygen, and carbon dioxide concentrations; a sensor placed in the tail detects the old culture medium; the injection process is adjusted according to the discharge liquid; and imaging technology monitors blood vessel development in organoids in real time. These features are more compliant with new *in vitro*, independent, specific, and controllable vascularized organoids pursued by scientists.

### Organoids Immune Environment

The immune system is an important system that performs immune response and immune function. This system comprises immune organs, immunocytes, and immune molecules, identifying and excluding foreign antigenic matter and coordinating with other body systems. Moreover, it maintains the stability of the environment and balances physiological processes within the body and is mainly involved in the development of various diseases. Traditional 2D cell lines can be co-cultured with different immune cells, but this is just a random mix and does not represent the communication between immune cells and other cells and does not characterize the immune microenvironment. If implanted in immunodeficient animals, xenografts limit the interaction between the host immune system and cancer cells. If implanted in immunocompetent animals, xenografts are easily mixed with inherent immune system cells of animals. In the following text, we will introduce the immune research of organoids from three aspects, including immune tissue construction, immune–epithelial interaction, and tumor immune microenvironment.

Since the development of organoids, scientists consider the thymus as the first immune organ. The thymus is a vital lymphoid organ located in the upper part of the anterior mediastinum behind the sternal stalk. Its function is to continuously produce various T-cell populations, which play essential roles in the body’s immune function. A study showed that transplantation of thymic organoids into non-thymic nude mice can effectively promote the homing of lymphocyte progenitor cells and support thymic hematopoiesis and immune response (Fan et al., 2015). Further, a recent study showed that combining epithelial–mesenchymal mixed cells with thymic interstitial cells and natural thymic acellular ECM can produce T-cells in humanized immunodeficient mice (Campinoti et al., 2020). These findings lay the foundation for cellular and molecular communications between ECM and thymocytes and provide a practical prospect for the construction of thymic organoids and thymus regeneration *in vitro*. These provide an opportunity to study tissue growth, development, and treatment of congenital and acquired immune diseases.

The interaction between epithelial cells and immune cells plays an essential role in the physiological balance in mammals. Integrating the interaction network of organoids, immune cells, and related cells is very important for tissue growth and development, establishment of related disease models, drug evaluation, pharmacological testing, and application in regenerative medicine.

**FIGURE 5:**
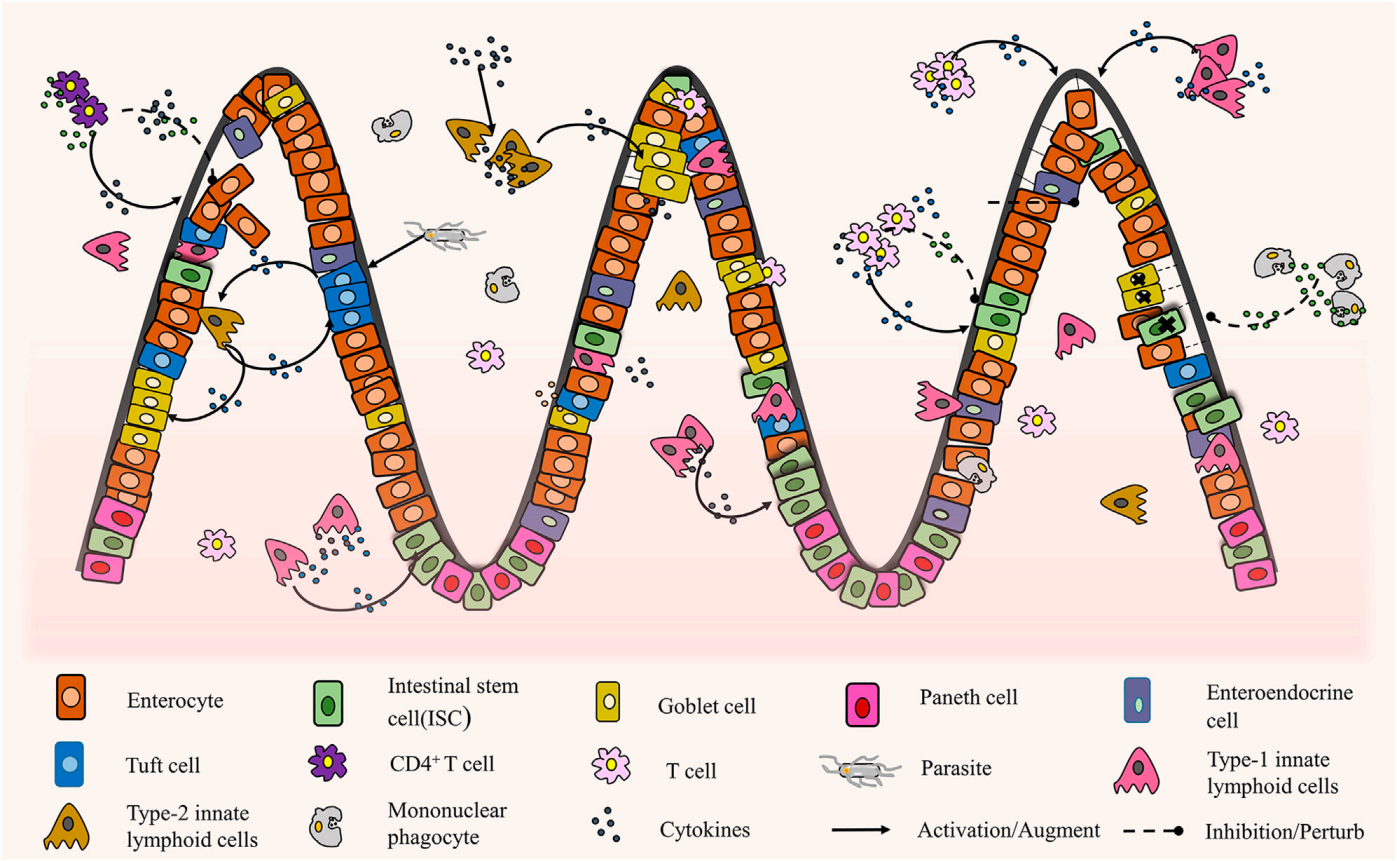
The interaction between intestinal organoids and immune cells (Schreurs et al., 2019; Howitt et al., 2016; von Moltke et al., 2016; Gerbe et al., 2016; Nozaki et al., 2016; Waddell et al., 2019; Lindemans et al., 2015; Biton et al., 2018; Takashima et al., 2019; Jung et al., 2018; Jowett et al., 2021; Ihara et al., 2018).

In this section, we will discuss the interaction between intestinal epithelial organoids and the surrounding immune environment. In the physiological environment of the small intestine, to maintain the dynamic balance of tissues, the intestinal epithelium regenerates continuously from ISCs to eventually differentiate into intestinal epithelial cells, Paneth cells, tuft cells, etc. Through the experiment using co-culture of organoids with immune cells and related cytokines, different levels of interactions were found between intestinal epithelial organoids and the surrounding immune environment *in vitro* (Figure 5). Different immune cells and their cytokines play diverse positive and negative roles in this dynamic tissue balance. The intricate interactions between them are the primary conditions necessary for preventing and curbing pathogenic infections and preventing excessive immunoreactive and subsequent tissue damage repair processes (Thaiss et al., 2016). Moreover, many studies are still using co-cultures of single immune cells and organoids, which are far from the complex immune microenvironment of the human body.

Immune cells are essential components of the tumor microenvironment and control cancer progression and drug response (Greten and Grivennikov, 2019). The emergence of tumor immunotherapy also revolutionized cancer treatment and inspired extensive research on tumor immunology and on using this “natural weapon” to gain therapeutic benefits. The problem of applying organoid technology to establish a model of the interaction between tumor and immune cells needs to be solved first. At present, two primary sources of autologous immune cells introduced into the organoids are available. The first source is the mixed tumor/lymphocyte organoids produced by introducing lymph node components or peripheral blood lymphocytes into the tumor organoids, such as pancreatic ductal adenocarcinoma (Holokai et al., 2020), melanoma (Votanopoulos et al., 2020), and colon tumor (Courau et al., 2019). Those methods model the tumor immune circulation, but most of them used epithelial-only organoids co-cultured with lymphocytes. The immune microenvironment of different tumors varies; even the same tumors have different immune environments. Therefore, a simple mixture of tumor organoids and immune cells cannot obtain the immune microenvironment and cannot be used in the experimental immunotherapy model. The second method is to construct the organoids with an immune environment. Scientists placed the tumor tissue in the air–liquid interface (Neal et al., 2018) or 3D microfluidic culture system (Jenkins et al., 2018), and the results showed that tumor spheres retain the original autologous matrix, related immune checkpoint deletion, tumor-infiltrating lymphoids, and myeloid subsets. This method retains the complex histological structures with tumor parenchyma and stroma, including functional tumor-specific tumor-infiltrating lymphocytes, which can effectively predict tumor development, and the effect of PD-1 or PD-L1 inhibition could be assessed (Neal et al., 2018). However, this method still has limitations, existing culture conditions cannot meet the needs of tumor cells, stromal cells, and immune cells simultaneously, and the unique tumor immune microenvironment will be gradually lost in the culture process. In the future, the development of tumor immune organoids will aid in the immune-oncology study of patient-derived tumor organoids in a tumor-preserving microenvironment. Tumor organoids with an immune microenvironment can aid in the analysis of the pathology between tumor and immune cells, reproduce cancer-related inflammation and canceration, screen and optimize cellular immunotherapy *in vitro*, and shorten the absolute distance between the laboratory and clinic.

In addition, tumor organoids with immune–vascular interaction have made a breakthrough (Cui et al., 2018). This organoid model can be used to culture tumor cells, endothelial cells, and immune cells from patients in a controllable microenvironment, which is applied to the phenotypic identification of various tumors and allows high-throughput drug screening and real personalized drug screening possible and advances precision medicine a step forward.

### Organoids Culture Medium

ECM is a complex hierarchical network composed of proteins, which affect a series of cell processes, such as adhesion, proliferation, and differentiation. The construction of biomaterials that mimic natural ECM characteristics is significant for studying cell physiology (Zhu et al., 2019). Similarly, in the construction of organoids *in vitro*, the selection and construction of culture medium are also critical. The construction and selection standards of all kinds of *in vitro* model culture matrices are focused on whether the model can better simulate human physiological and functional environment on a multi-scale from molecule to cell, tissue, organ, and even the whole organism. A hydrogel is a multiphase matrix composed of hydrophilic polymers with high water content, which has a highly porous structure similar to the natural ECM. Hydrogels are recognized as the first choice for simulating biological ECM to construct organoids *in vitro* because of their high biocompatibility, extraordinary permeability, appropriate elasticity, and hardness (Liu et al., 2019). According to their composition, hydrogels can be roughly divided into natural, synthetic, and hybrid hydrogels (Table 5).

**TABLE 5:** Organoids and hydrogels.

Category	Materials	Organoids	Features	Results
Natural hydrogel	Decellularized matrix	Airway organoids, liver, organoidsetc.	Native microstructure and extracellular matrix (growth factor, protein, etc.)	A viable approach for the generation of a whole functional organ Du et al. (2016), Tan et al. (2017), Vyas et al. (2018), Giobbe et al. (2019), Mollica et al. (2019), Rezaei Topraggaleh et al. (2019), Bi et al. (2020a), Bi et al. (2020b), Meran et al. (2020), Willemse et al. (2021)
	Nanocellulose fibers	Intestinal organoids	Well defined, low cost, and appropriate stiffness	Sustainable and promising microenvironment for organoid growth and budding Curvello et al. (2020), Curvello and Garnier (2021)
	Collagen IV (substrate) + Collagen I (overlay)	Intestinal organoids	Inter-lot stability, accessibility to the apical side, and scalability	Expanding IECs with higher LGR5+ mRNA levels Tong et al. (2018)
	Alginate	Intestinal organoids	Low-cost and controllable physical and biochemical properties	Supports human organoid growth *in vitro* and leads to epithelial differentiation Capeling et al. (2019)
	Bioactive glass + integrated alginate	HDF cells and KB3-1 cell spheroids	Porous and hardness increase, optimal swelling and porosity, and biocompatibility	Support the regeneration of bone, intra-vascularization, and neotissue formation Bargavi et al. (2020)
	Bone forming peptide-1 + silica nanoparticles + RGD + alginate	Osteo-differentiation of human mesenchymal stem cells	Uniform, additional mechanical integrity, suitable elastic modulus, and super-hydrophilic property	Enhance hMSC survivability, expansion, and osteogenesis and useful for tissue regeneration and 3D bio-printing Luo et al. (2018)
	Collagen	Thymic organoids and intestinal organoids	Biocompatible, biodegradable, easily detectable, and high porosity	Supports organoid growth, differentiation, structuralization, and a carrier *in vivo* Sachs et al. (2017), Bortolomai et al. (2019)
	Fibrin	Epithelial organoids	Enzymatic gelation and mimic signaling function	Supports the long-term expansion of all tested murine and human epithelial organoids Broguiere et al. (2018)
	Plasma	Breast cancer organoids	A reservoir for nutrients, growth factors, cytokines, and signaling molecules, and low cost	Supporting organoid growth and expansion and high-throughput drug screening and predicting clinically therapies Calar et al. (2020)
Synthetic hydrogel	QGel CN99	Colonic organoids	Fully defined hydrogel and freeze-dried preservation	Supports efficient establishment, expansion, and biobanking of primary human colonic organoids Bergenheim et al. (2020)
	Polyisocyanide + RGD (Arg–Gly–Asp)	Breast organoids	Biocompatibility, soft, stability, and thermal reversibility	Supports (cystic) organoid formation and maintains capacity to branch Zhang et al. (2020)
	Poly (ethylene glycol) (PEG) + RGD	Liver organoids and intestinal organoids	Controllable hardness and slow degradation	Optimized the efficiency of organoid derivation expansion and formation Gjorevski and Lutolf (2017), Sorrentino et al. (2020)
	PEG + integrin and matrix binder peptides	Intestinal organoids	A rigorously defined microenvironment and suitable storage moduli and swelling	Long-term growth of human organoids and broadly use for other epithelial organoid culture Hernandez-Gordillo et al. (2020)
	PEG + RGD + protease-degradablepeptide	Intestinal organoids	A regular mesh structure with fully swollen and symmetric blocks	Robustly supports HIO maintenance and an ideal injection vehicle Cruz-Acuña et al. (2017)
	Allyl sulfide	Intestinal organoids	With photocleavable bonds that temporally regulate the material’s bulk modulus	Provide more uniform and consistent organoid structures and particularly useful in drug screening Hushka et al. (2020)
	Amikacin	Pancreatic organoids	Tunable physiochemical properties	Significant enhancement of islet phenotype and self-organization into 3D spheroids Candiello et al. (2018)
Hybrid hydrogel	Poly (ε-caprolactone) + cancer-associated fibroblasts (CAF) ECM	Breast cancer organoids	Slow degradation kinetics, open, and well-connected porous architecture	Better mimic biochemical and biomechanical properties and capture drug response Nayak et al. (2019)
	Gelatin + chondroitin sulfate + hyaluronic acid (HA)	Retinal organoids	Stable and spongy with a homogenous compressive strength	Favored retinal cell types differentiation and weak immunogenicity Singh et al. (2018)
	Gelatin + phenol	Colorectal cancer organoids	Controllable hardness and smaller pore sizes with higher storage moduli	Support colorectal cancer organoid survival and drug sensitivity Ng et al. (2019)
	Matrigel + collagen I	Blood vessel organoids	Similar to natural ECM and stability	Generating blood vessel organoids and a model of diabetic vasculopathy Wimmer et al. (2019)
	PEG + fibrin	Lung adenocarcinoma organoids	Controllable mechanical properties, stability, and biocompatibility	Tumor responses reproduce and more accurately model solid tumors Del Bufalo et al. (2016)
	Hyaluronan (HA) + gelatin + CAF	Colorectal cancer organoids	Fully defined hydrogel and tunable biochemical and mechanical properties	Retained proliferative capacity and preserved molecular characteristics and CRC-CAF Luo et al. (2021)

IECs, intestinal epithelial cells; HDF, human dermal fibroblasts; hMSCs, human mesenchymal stem cells; ECM, extracellular matrix.

#### Natural Hydrogels

The emergence of tissue decellularization technology, coupled with the easy acquisition of animal tissue-specific ECM, makes it possible to use animal-specific natural hydrogels to culture human organoids. Matrigel, the most widely used commercial matrix, is a soluble basement membrane extracted from Engelbreth–Holm–Swarm mouse sarcoma and is rich in ECM proteins, such as laminin (main component), type IV collagen, and various growth factors. However, the composition of mouse sarcoma is unknown, and simulating actual conditions of healthy tissues in the microenvironment of tumor cells is difficult. Therefore, considering the unique ECM of different tissues, different organoids should be matched with the corresponding decellularized matrix. Renal organoids are constructed on decellularized renal matrix scaffold, and the pre-existing vascular and filtration systems of the matrix provide a natural conduit, promoting cell self-assembly and regaining of biochemical function (Du et al., 2016). Testicular organoids with spermatogenesis were constructed using a male goat testicular acellular matrix (Rezaei Topraggaleh et al., 2019). Thymic acellular matrix improved T-cell output microenvironment and construction of functional thymic organoids (Hun et al., 2017). ECM hydrogels from decellularized tissues can provide an environment that can guide cell growth and induce corresponding physiological functions. In addition, they have the proteomic characteristics of the source tissues and specific enrichment of significant ECM proteins related to organogenesis, which play crucial roles in regulating organoids. However, the decellularized matrix still has some limitations. There is a potential loss of soluble growth factors and cytokines in different acellular methods; even if the acellular technology is improved, we cannot eliminate such a loss. Animal-derived acellular matrix is at risk of species difference, immunogen, and pathogen transmission. The composition of ECM in different batches maybe is specific, which leads to the difficulty of performing accurate quantitative and qualitative analyses of the cultured matrix, so the experimental results are accidental. These persistent uncontrollable and potential nonphysiological physical environments seriously limit the use of acellular matrix and restrict the clinical and transformational applications of organoids *in vitro*.

Natural hydrogels have good biocompatibility, and scientists are pleased to find other suitable substitutes. For example, cellulose hydrogel is a kind of plant-based, sustainable, and low-cost soft material. Its mechanical properties are similar to those based on the animal matrix and are easy to be use. Cellulose hydrogel can induce the formation and growth of small intestinal organoids (Curvello et al., 2020). However, these natural hydrogels have relatively poor mechanical properties and insufficient stability, hindering the long-term cell culture in model systems. Hence, nanocellulose hydrogels cross-linked with divalent cations with ideal biochemical and physical properties (Curvello and Garnier, 2021) and alginate with enhanced properties of bioglass (Bargavi et al., 2020) or protein (Luo et al., 2018) are invented. Those new hydrogels have the potential to reduce the cost of biomedical research. Natural hydrogels are biocompatible and have mild physical and chemical properties. Furthermore, they provide biomimetic scaffolds that are rich in cell adhesion sites and wrap cells *in situ*, and they are easy to use. In the future, natural hydrogels will assist in the transformation and clinical application of organoids.

#### Synthetic Hydrogels

The dependence of some poorly defined natural sources of ECM severely limits the application and promotion of organoids on the large scale, prompting scientists to turn to chemical synthesis for help. The complete definition of the ingredients is a prerequisite for the large-scale use of hydrogels, which is critical to the stability and repeatability of the experiment. Synthetic hydrogels used in organoids include QGel CN99 (Bergenheim et al., 2020), polyisocyanide (Zhang et al., 2020), PEG (Cruz-Acuña et al., 2017; Gjorevski and Lutolf, 2017; Hernandez-Gordillo et al., 2020; Sorrentino et al., 2020), allyl sulfide (Hushka et al., 2020), and amikacin (Candiello et al., 2018). Fully defined synthetic hydrogels can be adjusted to have the desired physical properties (stiffness, porosity, elasticity, and absorption) by simply modulating the monomer mole ratio, synthetic microenvironment, or exposure duration (Candiello et al., 2018; Hushka et al., 2020; Sorrentino et al., 2020). Even after hydrogel synthesis, the physical and chemical properties are still controllable. Those properties are critical for biological activities such as expansion, contraction, migration, and differentiation of organoids. For example, solving the mechanical dependence on crypt structures is necessary for the construction of homogenous and reproducible intestinal organoids for clinical applications (Hushka et al., 2020) and elimination of the negative effect of abnormal liver mechanics on the proliferation of liver progenitor cells in organoids (Sorrentino et al., 2020). Avoidance of the adverse effects of hydrogels on organoids is an important part of the standardization of organoids in the future.

The outstanding advantage of synthetic hydrogels is that they reinforceable by adding various proteins. PEG is widely used in drug carriers and tissue engineering because of its unique physical and chemical properties and biocompatibility. RGD is a motif in many ECM proteins, including collagen and fibronectin, which can effectively promote cell adhesion to biomaterials. The combination of PEG and RGD can effectively promote the adhesion of cells to the gel and promote the formation and proliferation of organoids (Gjorevski and Lutolf, 2017) and, when combined with integrin and matrix binder peptides, strengthen epithelial cell proliferation and organoid formation (Hernandez-Gordillo et al., 2020). Furthermore, they can be combined with PEG, RGD, and protease-degradable peptide GPQ-W, which is more beneficial to matrix remodeling, cell migration, and growth (Cruz-Acuña et al., 2017). The quantitative and qualitative chemically synthesized matrix has the characteristics of adjustable key properties. Small molecules and soluble factors (such as growth factors) can be connected to hydrogels in a controllable and anisotropic manner through covalent interactions; thus, synthetic hydrogels are becoming more popular. The ECM in organisms has complex structure and composition, and even a small piece of tissue may have different structures and components. A single synthetic hydrogel added only with some factors still cannot meet the needs; hence, more research is desired to explore different structures and components to meet the needs of all kinds of organoids.

#### Hybrid Hydrogels

Given the complex environment in the human body, the use of a single type of hydrogel cannot meet all the requirements of constructing organoid models *in vitro*. Therefore, the hybrid hydrogel with better properties can be prepared by physical blending and chemical modification. The mixed synthetic matrix of natural hydrogels is biocompatible and avoids the disadvantages of some components. As a matrix for human intestinal epithelial cells, collagen I is more accessible to obtain (through various tissues or commercial sources). The ratio of collagen I to Matrigel (1:1) reduced the usage rate of Matrigel, cost, and time, in a sense, on the premise of ensuring the development of organoids (Wimmer et al., 2019). Alternatively, gelatin was used to prevent immunogenicity (Singh et al., 2018) or adjust mechanical strength and gelling rate (Ng et al., 2019; Luo et al., 2021). Natural–synthetic hydrogels may be the best choice, as they combined the advantages of natural and synthetic hydrogels. PEG–fibrin hydrogel forms tumor–stromal interactions and induces more invasive tumor organoids, with more advantages over other 3D systems (Del Bufalo et al., 2016). Fibrin hydrogels have good operation performance and biocompatibility, and changing the total concentration of fibrinogen can control the mechanical properties of these hydrogels. However, fibrin hydrogels are easily degraded by fibrinolytic enzymes and other enzymes, resulting in the uncontrollable and unpredictable loss of mechanical rigidity, low stability, and opacity. Therefore, the addition of PEG increases the stability and transparency of hydrogels. Similarly, poly (ε-caprolactone) provides an open, well-connected porous structure; decellularizing cancer-associated fibroblasts faithfully reflect the characteristics of ECM and promotes cell attachment and vitality (Nayak et al., 2019). They provide unique environmental and mechanical properties for primary cancer cells, thus improving the survival rate of organoids.

A series of processes such as adhesion, growth, proliferation, and differentiation were performed in the ECM. Natural, synthetic, and hybrid hydrogels have advantages and disadvantages, but they cannot fully simulate the tissue microenvironment *in vivo*, and cultural conditions are not entirely controllable. The above research also presents the best method of developing an ideal matrix in the future, including the following aspects: 1) composite hydrogels with various substances, such as bone-forming peptide-1, synergistically enhance the survivability, expansion, and osteogenesis of human mesenchymal stem cells (Sorrentino et al., 2020). Laminin-111 is a key parameter required for robust organoid formation and expansion (Broguiere et al., 2018). Additives enhance the contact between the matrix and organoids and enhance some properties of the hydrogel, which are more conducive to the formation and development of organoids. 2) The hydrogel has a structure similar to that of the environment *in vivo*, such as the decellularized matrix of homologous tissue (Giobbe et al., 2019; Mollica et al., 2019; Bi et al., 2020a; Bi et al., 2020b; Meran et al., 2020; Willemse et al., 2021), a culture hydrogel of intestinal organs composed of a collagen V-coated porous substrate, and a collagen I gel overlay (Tong et al., 2018); 3D collagen type I scaffolds mimicking the double cortical layer ultrastructure of the thymus (Bortolomai et al., 2019). Different tissues have different structures. The reconstruction of these structures is necessary to restore the structure and function of organoids *in vivo*, and it will also be the focus of regenerative medicine research in the future. 3) The combination of hydrogel and microfluidic platform forms organoids-on-a-chip, recapitulating the biochemical, mechanical, structural, and functional features of the human cellular microenvironment *in vivo*. The ECM *in vivo* is not a continuous layer but a dynamically balanced environment. Just as the ECM will be affected by body fluids, immune products, pressure, pH, oxygen content, and other factors, despite great technical difficulties, the microfluidic platform is also simulated on the hydrogel as much as possible.

## Conclusion

Organoids, cell lines, and xenografts have advantages and disadvantages. In experimental research, the most appropriate research model should be chosen according to the purpose, needs, and conditions. This review summarized recent research and advantages of current organoids in tumors, hereditary diseases, infectious diseases, and regenerative medicine, showing the infinite possibility of organoids as a new disease model. In addition, organoids have achieved success in tissue development (Huch and Koo, 2015), drug toxicology (Astashkina and Grainger, 2014), and neuropsychiatric diseases (Mariani et al., 2015). However, the shortcomings of organoids in vascularization, immune microenvironment and hydrogel still exist. Here we summarize and point out the development direction, just to provide reference for more researchers. In the future, organoids are expected to be continuously used in all fields of medical research, combined with various scientific and technological means, to constantly develop organoids’ potential. Although organoids have various limitations, with the application and improvement of organoids, the gap between its clinical applications will continue to reduce. As a result, it will be increasingly and directly used in the clinical treatment of patients, which has a broad and bright prospect.
